# Global Health: Shared Obligations and Mutual Respect

**DOI:** 10.5334/aogh.2539

**Published:** 2019-06-17

**Authors:** Lawrence O. Gostin

**Affiliations:** 1 O’Neill Institute for National and Global Health Law, US; 2 WHO Collaborating Center on National and Global Health Law, US

## Abstract

The commentary provides support for the article “Not Above the Law: A Legal and Ethical Analysis of Short-Term Experiences in Global Health” by Rowthorn et al. Gostin supports the paper’s assertion that anyone participating in health care activities in any setting (both at home and abroad) must abide by applicable laws and regulations. Engaging in such work without doing so reflects an outdated – and destructive – model of aid that presupposes and imposes an inherently unequal relationship that thwarts the goals and values of global health. He concludes that there can be no double standards and no ethical or legal values that apply in rich nations but not in countries that host health care volunteers.

*Ignorantia juris non excusat* (Latin: “ignorance of the law excuses not”) is an ancient legal principle holding that a person who is unaware of the law may not escape liability for violating it. In addition to eliminating a defense in court for unlawful behavior, the principle is also woven into our daily lives and social norms. As much as my charitable instincts may urge me to enter a hospital, school, or courtroom to help those I perceive as needing my assistance, I instinctively know that working in those settings must be done within the legal and regulatory frameworks built to protect patients, students, and clients. The law’s purpose is clear and compelling; regulatory systems for licensing or credentialing, for example, are aimed at ensuring that only those who have the requisite skills and character may practice in certain specialties.

I may not know the exact law that prevents me from caring for patients in an overcrowded emergency room, but I respect (or fear) the law sufficiently to channel my charitable instincts appropriately. This respect for the rule of law is the foundation of a civilized society and, when it works well, ensures adherence to principles that both liberate and protect. It is therefore axiomatic that, to respect and support the rule of law in other countries, visitors must adhere to the local laws and regulations that apply to their in-country activities. But well beyond law, social mores and ethical values tell us that to do good, we must have the requisite expertise – because without that expertise, we could easily cause harm, even if our motives were well-intentioned.

In this issue of the *Annals of Global Health*, a multidisciplinary team of scholars shines the light on a deeply problematic global practice, namely individuals from high income countries traveling to low- and middle-income countries (LMICs) to engage in clinical health care activities without inquiring about, or adhering to, local health laws and regulations. While some organizations and individuals certainly adhere to widely-available best practice guidelines for health-related engagement overseas, many others do not. Reports indicate that thousands of volunteers travel overseas for different reasons to engage in some kind of health care activity. Some volunteers are licensed health care professionals who engage in critical capacity building and humanitarian efforts; working in close partnership with local professionals and with regional health authorities. Others do not have the requisite skills or licensing credentials, often in violation of the laws in the host country.

Even if a volunteer were capable of acting helpfully in their country of origin, it is a different story when they travel to a LMIC. They have little, if any, training or experience to add value. Worse still, they could thwart local professionals or directly cause harm to patients themselves.

Volunteers may come with a variety of motivations: some genuinely well-intentioned and hoping to help; others for personal gain, such as putting it on their resume; and still others with motivations not rooted primarily in meeting the needs of local communities at all. Many scholars have carefully expressed the ethical concerns with “short term experiences in global health” (STEGHs). Rowthorn et al.’s article is the first to focus on the legal implications of certain STEGH practices, focusing on medical licensure and drug importation and distribution.

Rowthorn et al.’s article points out what is so obvious that having to say it highlights a disturbing complacency, ignorance, or simple arrogance on the part of some STEGH organizations and participants. Just as the United States has robust licensure and practice regulations, so do host countries. Although visitors may be unware of these laws, or find them administratively burdensome, these same considerations would never be an excuse to flout the law at home. And the fact that some volunteers perceive they can get away with it because LMICs supposedly don’t systematically enforce these laws is a shocking lack of respect for the host country and its population.

One has to ask – why would someone from the US, who by any metric would be considered a good and law-abiding citizen, travel to a foreign country and engage in activities that they know, or should know, are illegal in the US without asking if they are also unlawful in the country in which they volunteer? What tsunami of cultural, academic, and economic forces not only supports volunteers in such behavior, but encourages, even celebrates, it? I believe it is a vestige of an outdated model of charity that distorts the value and potential of the vital field of global health. In global health today, we don’t talk of “aid, assistance, or charity.” Instead we talk about mutual solidarity and joint responsibilities.

In my long career as a scholar at the intersection of law, ethics and global health, I have vigorously urged a vision of global health that values deep collaboration and partnership within and across nations to meet our shared obligation to improve the human condition. This vision rejects the outdated – and destructive – model of global health that is used as shorthand for health assistance provided by affluent countries to poor countries, in a donor-recipient relationship, as a form of charity. This flawed model of aid, that can be traced back in history to the colonial era and missionary excursions to remote areas, presupposes and imposes an inherently unequal relationship. In that skewed relationship, one side is a generous benefactor and the other a dependent. This ignores the complex long-term needs of LMIC health systems, populations, and laws. It also ignores the fact that all countries have a legal and ethical obligation, one to the other, for the global public good.

One of the greatest satisfactions of my career has been to witness the broad recognition – and success – of global health as an enterprise of collaborators sharing science and strategies across borders from north to south, south to north, and south to south. Whatever successes that have been achieved have been hard fought, through the greatest epidemics of our times, and based on decades of capacity building and cooperation. Today, we have so much to learn from, and share, with a corps of LMIC researchers and health providers who work for the public’s health within their own borders and well beyond. Supporting the idea of global health as a platform for multi-directional learning is also the recognition that the Global North has its own major deficiencies. Consider, for example, the broken US health system, which has the greatest global per capita cost, yet with weak outcomes. We too are gravely in need of low-cost, high impact interventions pioneered in low resource settings.

Notwithstanding this demonstrable shift in global health values, some vestiges remain, and I believe Rowthorn’s article describes one of them. While some organizations may believe that bypassing burdensome legal constraints is a necessary short-cut to meet the needs of underserved communities, Rowthorn and colleagues ably argue that this viewpoint is short-sighted and wholly inadequate in the long run for both volunteers or hosts.

The opportunity costs of creating clinical opportunities and experiences with US participants at the center instead of more sustainable, outcomes-oriented community-based initiatives is considerable and troubling. The recommendations in the paper are designed to encourage mutual respect and trust among citizens of different nations, which is the foundation of mutually beneficial and productive relationships. While health emergencies exist that will call for quick action, we still must insist that anyone participating in health care in any setting (both at home and abroad) must abide by host country laws, culture, and expectations. These legal and social norms are designed to safeguard the same patients STEGH programs purport to help. There can be no double standard, no ethical or legal value that applies in richer nations but not in host countries. We are all on this precious globe together and must have common aims and mutual respect for one another.

